# Microencapsulation of Gallic Acid Based on a Polymeric and pH-Sensitive Matrix of Pectin/Alginate

**DOI:** 10.3390/polym15143014

**Published:** 2023-07-12

**Authors:** Erik Francisco Nájera-Martínez, Elda A. Flores-Contreras, Rafael G. Araújo, Maricarmen Iñiguez-Moreno, Juan Eduardo Sosa-Hernández, Hafiz M. N. Iqbal, Lorenzo M. Pastrana, Elda M. Melchor-Martínez, Roberto Parra-Saldívar

**Affiliations:** 1 School of Engineering and Sciences, Tecnologico de Monterrey, Monterrey 64849, Mexico; erikfrancisco97@gmail.com (E.F.N.-M.); eldafc@tec.mx (E.A.F.-C.); rafael.araujo@tec.mx (R.G.A.); maricarmen.im@tec.mx (M.I.-M.); eduardo.sosa@tec.mx (J.E.S.-H.); hafiz.iqbal@tec.mx (H.M.N.I.); 2 Institute of Advanced Materials for Sustainable Manufacturing, Tecnologico de Monterrey, Monterrey 64849, Mexico; 3 Food Processing and Nutrition Group, International Iberian Nanotechnology Laboratory, Av. Mestre José Veiga s/n, 4715-330 Braga, Portugal; lorenzo.pastrana@inl.int

**Keywords:** phenolic acids, gallic acid, microencapsulation, biopolymers, alginate, pectin

## Abstract

The encapsulation of gallic acid (GA) through several methods has enhanced its shelf life and facilitated industrial applications. Polymeric matrices made of alginate and pectin were evaluated to encapsulate GA via spray drying. The pH-responsive release mechanism was monitored to validate the matrices’ performances as wall materials and extend the bioactive compound stability. The microcapsules produced were characterized via scanning electron microscopy (SEM), dynamic light scattering (DLS), Fourier-transform infrared spectroscopy (FTIR), and cyclic voltammetry (CV). The retention and encapsulation efficiency ranges were 45–82% and 79–90%, respectively. The higher values were reached at 3 and 0.75% (*w*/*v*) pectin and sodium alginate, respectively. The scanning electron microscopy showed smooth spherical capsules and the average particle size ranged from 1327 to 1591 nm. Their performance and stability were evaluated with optimal results at a pH value of 7 throughout the investigation period. Therefore, this work demonstrated the suitability of gallic acid encapsulation via spray drying using pectin and alginate, which are biopolymers that can be obtained from circular economy processes starting from agro-industrial biomass. The developed formulations provide an alternative to protecting and controlling the release of GA, promoting its application in the food, pharmaceutical, and cosmetic industries and allowing for the release of compounds with high bioactive potential.

## 1. Introduction

Reactive oxygen species (ROS), including superoxide radicals, hydrogen peroxide, hydroxyl radicals, and singlet oxygen, are by-products generated during the metabolism of biological systems. The appropriate synthesis and presence of ROS in cells are required for processes like protein phosphorylation, activation of transcription factors, apoptosis, immunity, and differentiation [1,2]. However, ROS have detrimental effects on proteins, lipids, and nucleic acids when their concentrations increase [1]. Studies showed that oxidative stress may have an important role in degenerative diseases (cancer, diabetes, metabolic disorders, atherosclerosis, and cardiovascular diseases). An alternative to modulating the concentration and activity of ROS is by raising the levels of antioxidant compounds [2]. One of the main proposals based on this fact is to increase the consumption of products with antioxidant activity. In line with this, phenolic compounds, which are a group of phytochemical compounds, are secondary metabolites and are known for their antioxidant properties. There are different types of phenolic compounds, such as phenolic acids, flavonoids, tannins, benzoquinones, and coumarins. One of the most important phenolic acids is gallic acid (GA) [3]. 

GA is a trihydroxybenzoic acid found naturally in many plants and fruits. It is particularly abundant in grapes; tea leaves (especially green and black tea); gallnuts; and certain berries, such as blueberries and strawberries. Also, it can be found in the aquatic plant *Myriophyllum spicatum* and the blue-green alga *Microcystis aeruginosa* [3]. GA has a strong antioxidant activity that helps to neutralize harmful free radicals in the body, protecting the cells from oxidative stress and damage. For this, GA was studied for its potential health benefits linked with its anti-inflammatory, anti-microbial, anti-cancer, and anti-diabetic properties. It may also have cardiovascular benefits and help in the treatment of certain skin disorders [4].

Even with the great properties and potential applications of GA, its use in the pharmaceutical and food industry is limited owing to its (i) astringent taste, where its strong taste may affect the flavor profile and consumer acceptability of the final product; (ii) pH sensitivity, where it can lose its antioxidant properties in alkaline conditions; (iii) stability, where it can be unstable and prone to degradation by exposure to heat, light, or oxygen; and (iv) potential interactions with other components. GA can interact with proteins, lipids, and other formulation compounds, which affects the functionality or stability of the product. These events can lead to a loss of its beneficial properties over time, affecting the shelf life of the products containing GA [3,5]. For these reasons, special formulation and storage considerations may be required to ensure its stability.

Accordant with this, several encapsulation techniques, including coacervation, micro- and nanoemulsions, ionotropic gelation, electrohydrodynamic process, and spray drying, have been evaluated to improve the stability and applications of GA in the industrial sector [3,5]. It is relevant to consider that spray drying has advantages over other techniques. For example, emulsion systems can be unstable over time, and due to the higher water activity, are susceptible to microbial contamination [5]. Furthermore, electrostatic gelation is a pH-dependent technique, which limits its use in the encapsulation of pH-sensitive compounds. In addition, the powders obtained by spray drying are regular in size and present few agglomerations; also, this is the method of choice for the industry based on its easy scaling-up and low operation cost [6]. Biopolymers are widely used as wall materials for encapsulation using this technique due to their high solubility, low viscosity at high concentrations, and low cost. Moreover, its incorporation in this kind of process promotes an alternative for the generation of added-value products into the concept of circular economy [7]. Among the main biopolymers used to encapsulate phenolic compounds are maltodextrin, pectin, alginate, starch, isolated whey protein, and Arabic gum [7,8,9,10].

Alginate is a linear anionic copolymer conformed by two monomers, namely, the α-L-guluronic and the β-D-mannuronic acids, and are attached by an *O*-glucosidic linkage [11]. Alginate is naturally present in brown algae in the form of alginic acid, being insoluble. The extraction of water-soluble alginate is made in the form of sodium alginate [12]. Meanwhile, pectin is a polysaccharide that represents nearly 35% of the cell wall in some plants and is mainly composed of galacturonic acid [13]. Pectin and alginate are biocompatible and biodegradable polymers with rheological properties that allow for their use as stabilizers, emulsifiers, and gelling agents in the food industry and for the microencapsulation of active compounds [14]. However, it was reported that alginate microcapsules obtained via spray drying are porous and dissolve quickly in water. An alternative to solve this drawback is reinforcement with pectin, increasing the stability of the microcapsules against different environmental conditions [15]. 

Alginate/pectin-based microcapsules have been designed mainly for the protection of compounds that are sensitive to moisture or light. This biopolymer combination has been used to encapsulate carvacrol under different inlet air temperatures via spray drying to determine the antioxidant and antimicrobial properties of the microcapsules. It was concluded that lower temperatures produced smaller, smoother, and spherical particles [16]. The functionality of alginate/pectin nano- and microcapsules was compared by considering the encapsulation of two essential oils and α-tocopherol via the ionic gelation method. The results allowed for the successful preparation of nanoparticles using ZnCl_2_ as a cross-linker, with yields of up to 70% and encapsulation efficiencies ranging from 60 to 80% [17,18]. To our knowledge, the development of microcapsules via spray drying that protect and enhance the performance and applications of GA for food and pharmaceutical sectors has been explored using a polymeric pectin/alginate mixture to validate its sensitivity to pH. In the field of microencapsulations, it is relevant to monitor the antioxidant properties in real environments, such as inside food formulations or in cellular compartments. The present project introduced CV to connect the encapsulation efficiency with the bioactivities [19,20]. Therefore, this study aimed for the encapsulation of gallic acid (GA) using pectin/alginate as the polymer shell via a spray-drying technique to create a hermetic reservoir for the compound and inhibit its degradation by environmental conditions.

## 2. Materials and Methods

### 2.1. Materials and Chemicals

The polymers, namely, sodium alginate from brown algae (CAS: 9005-38-3) and pectin from citrus peel (CAS: 9000-69-5), as well as substances such as the phenolic compound of gallic acid 97.5–102.5% (CAS:149-91-7), hydrochloric acid ACS reagent (CAS: 7647-01-0), Potassium chloride ACS reagent 99.00–100.5% (CAS: 7447-40-7), and ethyl alcohol pure ACS reagent 95% (CAS: 64-17-5), were obtained from Sigma-Aldrich^®^ (St. Louis, MO, USA).

### 2.2. Preparation of the Solutions 

The formulation of the biopolymeric solutions was made following the modified methodology of Sun et al. (2020) [16]. Five solutions were prepared individually based on weight/volume percentages (Table 1). The biopolymers were stirred at 100 rpm for 24 h to allow for their hydration in distilled water. Separately, the solution of GA was prepared at 1.7 mM according to the literature reports on the encapsulation of phenolic acids [21,22]. Then, the GA solution was added to the polymeric solution and mixed with a disperser (IKA- T10 Basic Ultra Turrax, Ika- Labortechnick, Staufen, Germany) at 2000 rpm for 5 min. Finally, the mixture was sonicated at 28 kHz (Digital Sonifier^®^ Unit, Model S-150D, Branson Ultrasonics Co., Danbury, CT, USA) for 5 min at 4 °C.

### 2.3. Microencapsulation of Gallic Acid on the Polymeric Solutions 

The microcapsules were dried in a Yamato spray dryer (Model ADL311S-A, Tokyo, Japan). The inlet and outlet temperatures used were 120 and 60 °C, respectively. The spray air pressure was set to 0.2 MPa. The sending pump flow rate was about 5 mL/min, and the diameter of the nozzle tip hole was 0.2 mm. These conditions were applied to all the microencapsulation processes. After the development of the microcapsules, the percentage of synthesis yield was calculated by dividing the weight of the microcapsules recovered after the spray-drying process by the total weight of the mixture (GA and formulation based on alginate, pectin, or a combination of both) multiplied by 100:

$$Yield\;of\;synthesis\;\left(\%\right)=\frac{W_{M}}{W_{C}}\times 100$$

where $W_{M}$ and $W_{C}$ are the weight of microcapsules after the spray drying process (g) and the total weight of the solids and GA in the feed solution (g), respectively. Microparticles were obtained in triplicate. 

### 2.4. Characterization of the Synthesized Microcapsules

The microcapsules were characterized via spectroscopic and microscopic methods to determine their morphology, size, and stability.

#### 2.4.1. Characterization Using Fourier-Transform Infrared Spectroscopy (FTIR) 

To analyze the composition and linkages in the microcapsules resulting from the spray-drying process, the FTIR technique was used. Each batch of microcapsules was analyzed using a Frontier spectrometer (PerkinElmer, Waltham, MA, USA) equipped with the universal ATR sampling accessory to obtain the FTIR of the microcapsules. The spectra were obtained in the range of 400 to 4000 ${\mathrm{cm}}^{-1}$ with 64 scans and a resolution of 4 ${\mathrm{cm}}^{-1}$.

#### 2.4.2. Characterization Using Dynamic Light Scattering (DLS) 

The size distribution of the microcapsules was analyzed using a Zetasizer Nano ZS (Malvern Instruments, Malvern, UK). The absorbance and refractive index of the microcapsules were obtained to correctly measure the size distribution, polydispersity index (PDI), and zeta potential. For this, 20 mg of microcapsules were suspended in pure ethanol, stirred, and sonicated briefly to avoid aggregates. Then, the sample was diluted with water in a 1:2 proportion and measured in the equipment at 633 nm and 25 °C. The test was carried out for each nanoparticle formulation.

#### 2.4.3. Characterization Using Scanning Electron Microscopy (SEM) 

The morphology of the microcapsules was studied using a scanning electron microscope (Zeiss, EVO MA25, Overcoached, Germany). Before the analyses, the microcapsules were immobilized in a double-sided carbon tape and covered with gold with the aid of an ionic cover. The microcapsules were seen with an accelerating voltage of 10 kV under a high vacuum and with a magnification of 5000×.

#### 2.4.4. Cyclic Voltammetry Study

Microcapsules with high encapsulation efficiencies were chosen to study their electric properties via CV. The intensity of the signal given by the equipment can directly correlate with the concentration of GA in the microcapsules using the principle of the Randles–Sevcik equation for reversible systems. In this case, the peaks represent the positive currents from oxidation. 

For the voltammetry tests, three buffers were prepared at pH values of 7 and 14. Reactants of oxalic acid ($C_{2}H_{2}O_{4}$), potassium chloride (KCl), and potassium hydroxide (KOH) were used, respectively. Two measurements were made for this study, namely, with 20 and 200 mg/mL of each microcapsule, which were batch mixed with each pH buffer as a separated solution. The measurements were carried out by depositing 50 μL of the mixture in a screen-printed electrochemical cell and analyzed with a scan speed of 50 mV/s and a work window of [−0.4,1] V. Each measure was made twice to ensure the reliability of the electrochemical cell. The diameter of the screen-printed electrochemical cell was 3 mm, the reference material was the Ag/AgCl electrode, and the counter electrode material was carbon (EmStat4S potentiostat/galvanostat, PalmSens BV, GA Houten, The Netherlands).

### 2.5. Evaluation of the Retention and Encapsulation Efficiency

The calculation of the retention and encapsulation efficiency was undertaken according to the modified methodology of Sun et al. (2020) [16]. The retention efficiency refers to the GA present in the microcapsules after the encapsulation in comparison with the total GA added to the biopolymeric solution. Meanwhile, encapsulation efficiency refers to the total GA encapsulated minus the GA present on the surface of the microcapsule from all the GA present in the microcapsules.

For this determination, samples of 20 mg of each batch of microcapsules were first washed with pure ethanol. The supernatant was recovered and analyzed in a UV-Vis spectrophotometer (DR 5000 Hach Spectrophotometer, Loveland, CO, USA) at 280 nm. Then, the washed microcapsules were dissolved in water for 1 h under constant stirring and read using a UV-Vis spectrophotometer. The standard curve was prepared by dissolving pure GA in water using concentrations in a range from 0.0125 to 1.7 mM. The equations used to calculate the retention and encapsulation efficiencies are listed below in Equations (1) and (2), respectively. The test was carried out in triplicate and repeated twice.

(1) $$Retention\;efficiency\;\left(\%\right)=\frac{Total\;gallic\;acid\;in\;microcapsules\;\left[mM\right]}{Total\;gallic\;acid\;added\;\left[mM\right]}\times 100$$

(2) $$Encapsulation\;efficiency\;\left(\%\right)=\frac{Total\;gallic\;acid\;in\;microcapsules\;\left[mM\right]-Gallic\;acid\;in\;microcapsules\;surface\left[mM\right]}{Total\;gallic\;acid\;in\;microcapsules\;\left[mM\right]}\times 100$$

### 2.6. GA Release Kinetics of the Microcapsules

The studies of the release kinetics of the microcapsules were performed to evaluate their performance regarding the ending application.

Solutions at 4, 7, and 10 pH values were prepared using HCl, KCl, and KOH, respectively. Then, 40 mg of each microcapsule batch was placed in each one of the different pH solutions and incubated at 25 °C under constant stirring for 6 h. Aliquots of 200 µL were taken and measured every hour at 280 nm in the UV-Vis spectrophotometer (DR 5000 Hach Spectrophotometer). The release profile values were obtained using Equation (3) presented below. The test was carried out in triplicate and repeated twice.

(3) $$Release\;profile\;\left(\%\right)=\frac{measured\;gallic\;acid\;\left[mM\right]}{Initial\;gallic\;acid\;\left[mM\right]}\times 100$$

### 2.7. Statistical Analyses

The data were processed using one-way analysis of variance (ANOVA). The statistical data analysis was performed using the software Statistica v.10. (StatSoft Inc., OK, USA). The post hoc Tukey’s test (*p* ≤ 0.05) was used for the means comparison.

## 3. Results and Discussion

### 3.1. Microcapsules Characterization

All the developed formulations provided white microcapsules. The characterization of the microcapsules produced included spectroscopic, microscopic, and voltammetric studies. The results obtained are shown in the subsections below.

#### 3.1.1. FTIR Analysis 

**Figure 1:**
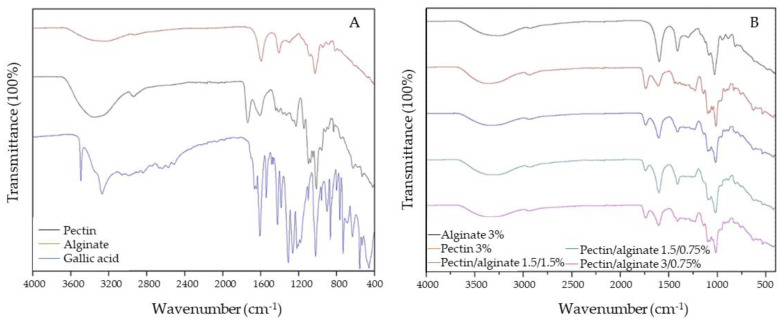
FTIR spectra of the (**A**) materials used in the microencapsulation and (**B**) microcapsules obtained with the different formulations.

The spectra shown in Figure 1B correspond to each one of the microcapsules produced in this study. In general, specific functional groups were evidenced, such as the characteristic O-H near 3400 ${\mathrm{cm}}^{-1}$, as this bond is present naturally in alginate and pectin. At 2920 ${\mathrm{cm}}^{-1}$, a small signal can be seen; this peak corresponds to the C-H vibration found in methyl groups.

Another peak that all the microcapsules produced had in common was asymmetric and symmetric ${\mathrm{COO}}^{-}$ stretching located at 1598 and 1410 ${\mathrm{cm}}^{-1}$, respectively, which correspond to the D-manuronic and L-guluronic groups present in the alginate.

All the microcapsules, except the alginate capsules, showed a peak at 1746 ${\mathrm{cm}}^{-1}$ representing a C=O bond, which was reported in other microcapsules formed using alginate and pectin, and is caused by the addition of the stretching C-O of the carboxyl group in pectin [23]. In addition, all the microcapsule batches showed a peak that was located at 1033 ${\mathrm{cm}}^{-1}$, which corresponds to the C-O-C stretching vibrations. In general, the spectra from the microcapsules were similar to those of the pure substances (Figure 1A); this was due to a lack of interaction between the biopolymeric matrices with the GA. Any signal of GA was registered, suggesting that the bioactive compound could be easily released from the matrix due to the lack of the development of linkages. These results are in agreement with the work of Vallejo-Castillo et al. (2020) [24], who showed similar results using a mixture of alginate and pectin to encapsulate GA via in situ and two-step entrapment methods [24].

#### 3.1.2. DLS Analysis and Synthesis Yield

The results registered by the DLS characterization of the microcapsules were recorded in the function of their diameter size in nanometers (nm). The condensed results are shown in Table 2. The low diameter of the microcapsules can be related to the use of the ultrasonic treatment, which decreased the droplet size through two mechanisms: (1) the acoustic field produced interfacial waves to break the dispersed phase into the continuous phase and (2) the formation of acoustic cavitation was used to collapse microbubbles into droplets of micro- or nanometric size by pressure fluctuations [15].

Other studies that used alginate or pectin as wall materials reported similar sizes for the microcapsules obtained; for example, Bastos et al. (2020) [25] obtained alginate (a concentration of 0.1%) particles of a size of 1731 nm to encapsulate black pepper essential oil by coacervates of β-lactoglobulin/sodium alginate for its use in the food industry. Furthermore, Hartini et al. (2021) used pectin extracted from jelly fig as the wall material to microencapsulate curcumin via the spray drying method for its application in the food and pharmaceutical industries [26], obtaining particle sizes ranging from 1390 to 2610 nm. Compared with the reported investigation by Sun et al. (2019), the phenolic compound carvacrol was protected in microcapsules made of pectin and alginate at 3% (*w*/*w*), obtaining particles with sizes of 1000 to 3000 nm, which agreed with the present work [27]. However, there were studies that considered a bigger particle size; for example, the work carried out by Norcino et al. (2022) [28], in which they generated microcapsules of 17,100 nm made from alginate and 21,900 nm from pectin to encapsulate anthocyanins via the ultrasonic gelation technique for its implementation in the food industry. These variations in the sizes of the microcapsules could be based on the techniques used for the development of the particles, as well as the different ratios of alginate and pectin. Even so, the increased percentage of pectin in the formulations was associated with increasing the volume of the microcapsules [29], which was consistent with those observed in Table 2.

**Figure 2:**
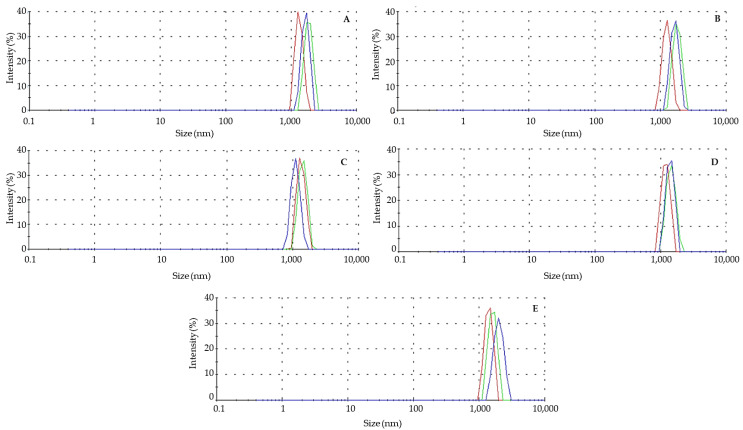
Particle size distributions for gallic acid nanoparticles obtained at different polymer concentrations: (**A**) alginate 3%, (**B**) pectin 3%, (**C**) pectin/alginate 1.5/1.5%, (**D**) pectin/alginate 1.5/0.75%, and (**E**) pectin/alginate 3/0.75%. Each lines of different color represents the obtained measure for each sample at the corresponding polymer concentration.

Monopolymer and composite nanoparticles showed a similar profile distribution. In both cases, the nanoparticle size distribution curves displayed comparable single peaks, showing that the particle dispersion was relatively concentrated (Figure 2). Similar results were previously reported for black pepper oil in β-lactoglobulin/sodium alginate nanocapsules [25], and curcumin in pectin microcapsules [26]. The PDI is a measurement of a sample’s size-based heterogeneity. This distribution pattern is expressed using values ranging from 0 to 1. The International Organization for Standardization (ISO; ISO 22,412:2017 and ISO 22,412:2017) determined that monodisperse samples are more likely to have PID values below 0.05, whereas polydisperse samples will show values above 0.7 [25,28]. In this research, the PDI values ranged from 0.12 to 0.83 (Table 2), which agreed with previous reports concerning the use of natural polymers in the encapsulation process [25,26]. These values were attributed to the development of aggregates due to the electrostatic interactions between the polymers. Moreover, the particle sizes obtained in the different formulations in this study were concentrated in a broad monomodal distribution [25,27]. The strongest indicator for the stability of dispersions is the zeta potential, which measures the electrostatic attraction or repulsion between particles [24,26]. The zeta potential of the microparticles obtained in this research was significantly lower at a ratio of 1:1 pectin/alginate (21 mV), suggesting that was the most stable system [24]. Zeta potential values between −30 mV to +30 mV are considered to have sufficient repulsive force to attain better physical colloidal stability [27,29].

The yield percentages of the synthesis of microcapsules ranged from 21% to 81.33% (Table 2). The lower yield was obtained for alginate-based microcapsules. This was explained based on the gelling of the alginate before pulverization by the spray drying technique, which was already reported in previous studies to be the main variable that modifies the synthesis yields [30]. On the other hand, the pectin-based microcapsules presented a percentage of 81.33% (with an inlet temperature of 120 °C). Compared with the literature, this was higher than the synthesis yield ranges of 56.04 to 70% reported by Hartini et al. (2021) [26] for pectin microcapsules using inlet temperatures of 80–90 °C. These comparisons indicated that the inlet air temperatures influenced the synthesis yield. Low temperatures produce microcapsules with higher humidity; in contrast, high temperatures could result in a high evaporation rate of the water. In both cases, the powder adheres to the drying chamber, increasing the losses. Moreover, the kind and proportion of material wall used can also affect the yield [31].

#### 3.1.3. SEM Analysis 

**Figure 3:**
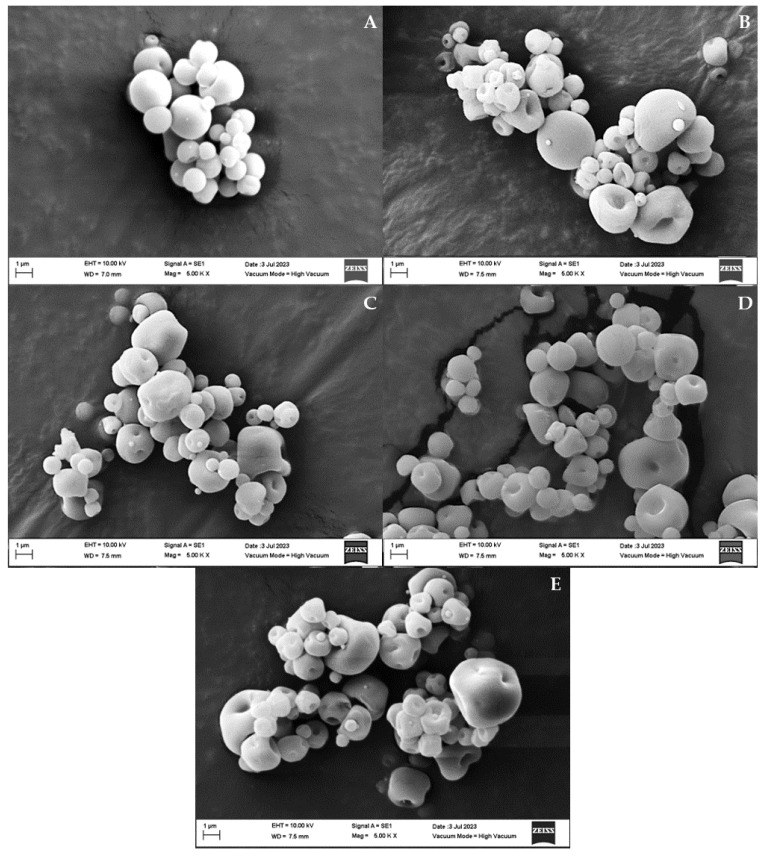
SEM micrographs of the gallic acid microcapsules produced at different polymer concentrations: (**A**) alginate 3%, (**B**) pectin 3%, (**C**) pectin/alginate 1.5/1.5%, (**D**) pectin/alginate 1.5/0.75%, and (**E**) pectin/alginate 3/0.75%, magnified by 5000×.

SEM studies revealed that the use of spray drying to encapsulate GA using pectin and alginate as wall materials allowed for obtaining spherical microcapsules with a smooth surface. The presence of particles of different sizes in the SEM results agreed with the PDI values (Table 2), which suggested that the obtained formulations were formed using particles of different sizes in a monomodal broad distribution [25,28]. Similar results were reported by Carlan et al. (2019) [32], who synthesized microcapsules of alginate and pectin via the spray-drying technique to carry vitamin B. The morphological properties are a consequence of the encapsulation agent, which determines the size and shape that the microcapsules will acquire. Depending on the biopolymer used, deformed spherical microcapsules with a rough surface (Arabic gum and maltodextrin biopolymers) or with an irregular shape and without a surface (xanthan and modified starch biopolymers) can be obtained [32]. On the other hand, Figure 3 shows the flattened form of a small population of microcapsules, which may be a consequence of general operating parameters of the spray-drying equipment, such as the inlet and outlet air flow temperatures, the feed flow, and the evaporation rate of water. Furthermore, the vacuum generated during the SEM analyses can also contribute to the presence of this form [33] However, the exact mechanism by which these parameters affect the morphology of the microcapsules is still unknown. The spray-drying technique is based on trial and error and it was observed that the higher the evaporation rate, the smoother the walls of the microcapsules [32,34]. 

In general, the microcapsules produced by the formulations presented in Table 2 did not have cracks or fractures on their surface, which meant an optimal interaction between the combined biopolymers that was enough to endure the expansion and swelling phenomena they are subjected to during the spray-drying process. In addition, the microcapsules were protected against air permeability, prolonging their stability over time [33]. At the same time, the micrographs showed the presence of agglomerated microcapsules in all formulations; this can be explained as a consequence of the electrostatic interaction generated between particles [35]. Furthermore, it is important to emphasize that the microcapsules did not present GA particles attached to their surface (Figure 3), which is an indicator of an efficient encapsulation of the GA inside the wall of the microcapsules [36].

#### 3.1.4. Cyclic Voltammetry 

**Figure 4:**
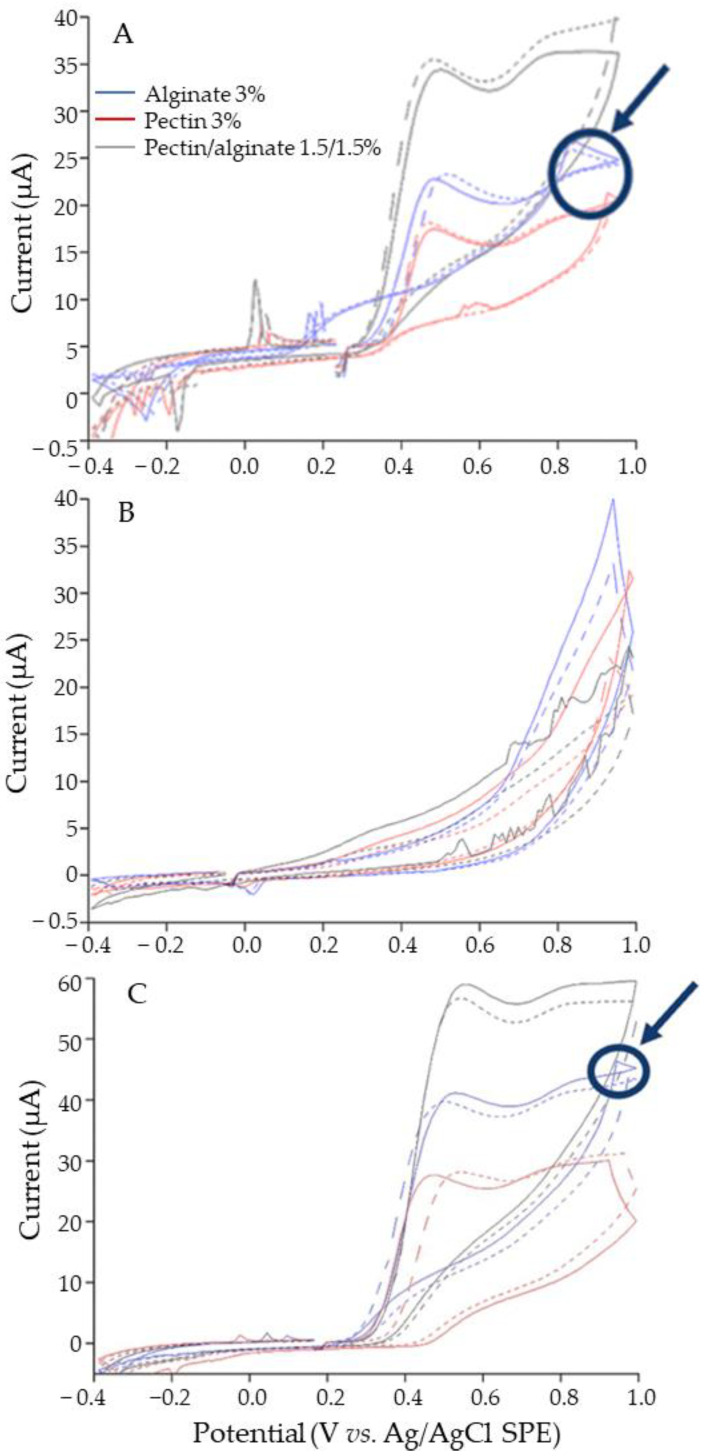
Cyclic voltammetry of the pectin/alginate microcapsules tested in pH 1, 7, and 14. The scan speed used was 50 mV/s over a screen-printed cell of diameter 3 mm. (**A**) Analysis of the microcapsules at a 20 mg/mL concentration and pH 7. (**B**) Analysis of the microcapsules at a 20 mg/mL concentration and pH 14. (**C**) Analysis of the microcapsule at a 200 mg/mL concentration and pH 7. Voltammograms solid and dashed curves represent the measure of one sample and its replicate, respectively. Circles and arrows indicate a possible sample contamination.

CV is a technique that is widely used for the detection of GA in different matrices. However, it is widely used to measure the antioxidant potential of compounds, as it serves as a reference expressed in equivalents of GA. The results obtained via CV are presented in Figure 4. The microcapsule formulations tested via CV were the individual biopolymers (pectin 3% and alginate 3%) and the mixture of (pectin/alginate at a 1:1 ratio); these formulations were chosen due to their high encapsulation efficiencies and the opportunity to compare the effect of the wall material in the voltammetry measures. 

The results of the microcapsules tested at pH 7 showed an oxidation peak that belonged to the encapsulated GA and there was no significant interference caused by the biopolymers used as wall materials (Figure 4A). The microcapsules tested at pH 14 (Figure 4B) did not show the proper GA signal that was present in the neutral pH medium, which could mean that the microcapsules were dissolved fast in the alkaline medium and the released GA was left unprotected and degraded. In addition, the release kinetics studies supported the lower dissolution time of the microcapsules and the fast rate of GA release into the medium. 

The maximum current was reached by the pectin/alginate 1.5:1.5 ratio microcapsules at both the 20 and 200 mg/mL concentrations. Different microcapsule concentrations were measured to determine whether there was a relationship between the intensity read and the quantity of gallic acid present in the microcapsules [37]. Compared with the results obtained for the encapsulation efficiencies shown below, the formulation made of pectin/alginate 1.5/1.5% had a higher encapsulation efficiency, which suggested there was a higher concentration of GA available inside the microcapsules. The concentrations of 20 and 200 mg/mL showed a small signal on the right side of the diagram; this observation is emphasized by the small circles and arrows in Figure 4A,C and might have been caused by failures in the connection or minor contaminations in the sample. 

A higher concentration of 200 mg/mL was used with the expectation that an increase in the concentration could increase the current reported by the voltammetry spectrum. An increase in current from 12.5 to 60 μA was confirmed with an increase from 20 to 200 mg/mL. However, the signal observed did not increase proportionally to the number of microcapsules studied, which would have led to an increase from 12.5 to 125 μA. This could have been a consequence of the saturation of the solution and an apparent high density and resistance to the flow. 

Studies that used CV to identify GA have been performed. For example, Zha et al. (2022) [38] used CV to detect GA using a bimetallic organic framework, demonstrating that CV is a technique that is able to estimate the real concentration of GA in a sample. In addition to this, CV is a powerful technique that enables the establishment of conditions for the delivery of antioxidant compounds. For example, using CV, it was possible to establish that the optimal temperature for the delivery of proanthocyanidins from zein nanofibers was 25 °C, and due to the increase in temperature, the oxidation peak disappeared. This implies the loss of the antioxidant properties of the active compound [19]. Hence, CV is a useful technique for studying the electrochemical behavior of phenolic compounds in delivery systems and helps to optimize the delivery of phenolic compounds, such as GA, to control and predict the rate of release.

### 3.2. Retention and Encapsulation Efficiencies 

The encapsulation efficiency is a consequence of the quality of the microcapsules produced and the level of protection offered by the biopolymers to the GA trapped inside the microcapsules [39]. The highest retention and encapsulation efficiencies were obtained with formulation 5 according to Table 3. Meanwhile, the lowest retention and encapsulation values were observed for formulation 1, corresponding to pectin/alginate at 3/3%. Additional reports in the literature revealed the protection of natural compounds by microencapsulation using a spray-drying technique. Sun et al. (2020) obtained encapsulation efficiencies of 75.64 and 55.51% for tangeretin at concentrations of 0.2% and 2%, respectively; the retention efficiencies were 71.05% (0.2% tangeretin) and 98.2% (2% tangeretin) using a combination of 3% (*w*/*v*) pectin and alginate. It seems that the retention efficiency could vary with the temperature of the emulsion during the spray-drying and homogenization processes [16].

Sun et al. (2020) [16] reported a pectin and alginate formulation to protect carvacrol, where they obtained retention efficiencies of 88.69, 48.78, 19.21, and 10.05% using inlet air temperatures of 100, 130, 160, and 190 °C, respectively. The authors concluded that temperature was a relevant factor in the protection of the volatile fraction and bioactive properties of natural compounds [16].

Moreover, the properties of pectin and alginate also play an important role in the efficiency of encapsulation and retention. In general, formulations made with 100% pectin present a poor protection barrier, decreasing its encapsulation efficiency and promoting its rapid release. The microencapsulation produced using 100% alginate was very narrow, decreasing the retention and encapsulation efficiencies as a result [29]. 

### 3.3. Release Kinetics

**Figure 5:**
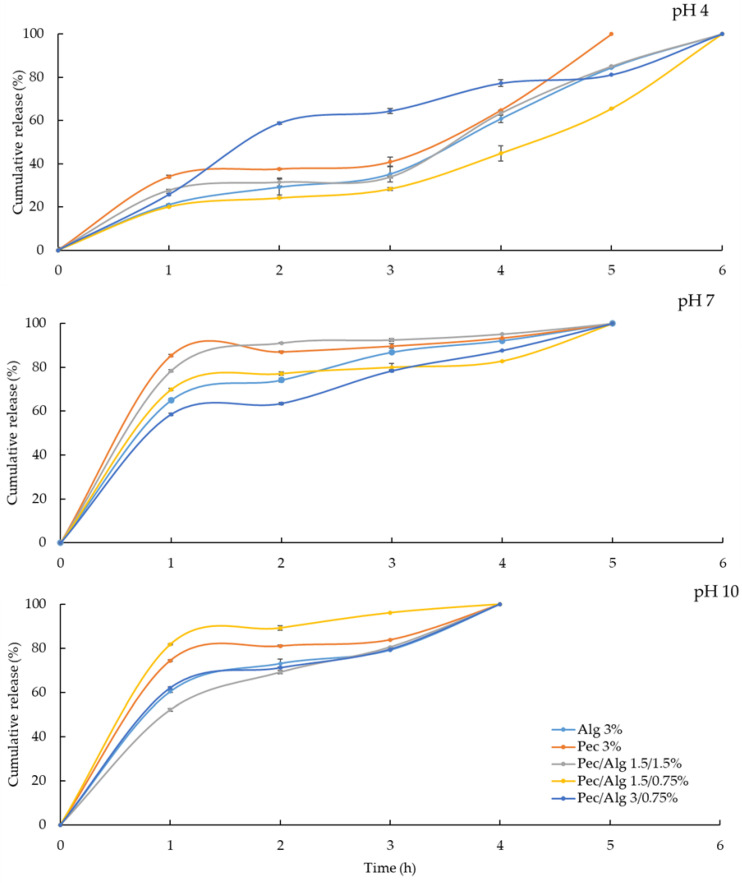
Kinetics of gallic acid release from pectin/alginate microcapsules in solutions at different pH values.

The release kinetics of the microcapsules was tested at 4, 7, and 10 pH values (Figure 5). The microcapsules tested at pH 4 showed a stable behavior over time compared with the microcapsules tested at neutral and alkaline pHs. The combination of pectin and alginate resulted in an attractive solution for the gradual and cumulative release in an acidic environment. Madziva et al. (2005) [40] obtained similar results using a system of alginate/pectin at a 70:30 ratio using calcium chloride as a crosslinking agent (0.1 M). At an acidic pH (1.2), they observed a cumulative release of folic acid around 20% after 144 min. Regarding an alkaline condition at pH 8.2 for a time of 80 min, the microcapsules released around 90% of the folic acid [40]. It is relevant to mention that the pectin used in the present study had a high percentage of methoxylation, which promoted the interaction with the guluronic acid residues in alginate throughout the formation of hydrogen bonds between the methoxyl groups and the hydroxyl groups. The decrease in pH favored the stability of the microcapsules because of the hydrogen bonding and it did not alter the hydrophobic interactions and attractive forces on the structure biopolymers [41]. This was observed in the cumulative release at pH 4, where all formulations, except pectin, were dissolved completely by 5 h. In contrast, the microcapsules at pH 7 were completely dissolved by 5 h as a consequence of the reduction in the $H^{+}$ ion concentration. Finally, the microcapsules at pH 10 were dissolved completely after 4 h of the experiment. The historical cumulative release percentages were in the ranges of 15–35%, 55–85%, and 50–85% at pH values of 4, 7, and 10, respectively, during the first hour. Alginate and pectin polymers have synergistic properties that allow them to keep uncommon microstructures distinct from each other. Their applications can cover an extensive range of compound protection, for example, essential oils, phenolic acids, and different natural actives for pharmaceutical and food incorporation [8,16,17]. This study did not measure the moisture content on the synthesized microcapsules, but it was reported that microcapsules prepared with crosslinked ions showed homogeneous behavior and lower moisture content. According to this, the divalent ions form a matrix that permits higher water diffusion and evaporation. An extensively used practice to control the surface area is adding maltodextrin to the polymeric mix because it allows for higher water diffusion from the core to the outside of the spray-dried particle. Otherwise, microcapsules prepared under a scheme using crosslinking ions present the largest particle size; therefore, they may form larger aggregates, resulting in lower water diffusion [17].

Moreover, the use of calcium chloride as a crosslinking agent can be an alternative to control the release of the encapsulated compounds. Polymers, such as alginate, increase the stability of the formulation by adding a cross-linking agent, such as the metallic ${\mathrm{Ca}}^{+2}$ ions. These ions promote the formation of the “egg box” structure, which makes these polymers resistant to dissolution due to the linking of the galacturonate and guluronate blocks [42]. However, even with the use of crosslinking agents, such as calcium chloride, the release of the active compounds will be strongly affected by the pH of the solvent media [40]. In line with this, the use of different concentrations of calcium chloride has not shown a significant impact on the release control. After 3 h, 75 and 90% of the encapsulated serum bovine released alginate microcapsules using calcium chloride at 1.2 and 2.4%, respectively [7]. Hence, all these results suggest that sodium alginate can provide control of the release of active compounds in products with a low pH.

## 4. Conclusions and Future Recommendations

The properties of phenolic acids and antioxidant compounds can be improved via encapsulation in polymeric matrices. Even with the wide range of methods for obtaining and extending the shelf-life of sensitive compounds, several conditions establish the spray-drying technology as a successful method to generate pH-sensitive microcapsules for use as carriers of antioxidant compounds. In addition, an extensive catalog report of polymers for making microcapsules is presented in the literature. In the food industry, pectin is represented as an available exemplar that is recovered via the circularity of agro-industrial waste, for example, from citric waste, coffee pulp, and other fruit waste. Alginate has been used as an emulsifying and stabilizing agent to coat fruits and vegetables. In this work, combining both polymers allowed for the encapsulation of phenolic acids with high retention efficiency (83%) and release efficiency (90.35%) when pectin/alginate 3/0.75% was formulated. Moreover, the best behavior was observed at pH 4 for a long time of 5 h, evidencing the stability of the wall materials related to the chemical nature of the polymers and their proportions in the formulation. Based on this fact, the microcapsules obtained may be a successful candidate for the protection of phenolic compounds and their potential use in the food industry, contributing to trends in the use of natural compounds to extend the shelf-lives of comestibles. Based on these findings, cyclic voltammetry can be a simple method to monitor the release of bioactive compounds in a complex matrix of substances on a lab scale or application in the field; moreover, for large-scale applications, additional techniques with a wide range of opportunities have been used in the industrial sector for years.

## Figures and Tables

**Table 1:** Formulations of the biopolymeric solutions used for microencapsulation.

Batch Number	Formulation	Polymer Concentration (*w*/*v* %) *
1	Alginate	3
2	Pectin	3
3	Pectin/alginate	1.5/1.5
4	Pectin/alginate	1.5/0.75
5	Pectin/alginate	3/0.75

* Weight/volume percentages.

**Table 2:** DLS characterization and synthesis yield of microcapsules made from sustainable biopolymers.

Batch Number	Formulation	Biopolymer Ratio (*w*/*v* %)	Z-Average Size (nm)	Zeta Potential (mV)	PDI
1	Alginate	3	1476.00 ± 189.69 ^a^	46.67 ± 5.17 ^d^	0.35 ± 0.04 ^c^
2	Pectin	3	1559.00 ± 350.30 ^a^	81.33 ± 3.56 ^a^	0.46 ± 0.10 ^bc^
3	Pectin/alginate	1.5/1.5	1327.33 ± 272.43 ^a^	21.00 ± 4.19 ^c^	0.63 ± 0.06 ^ab^
4	Pectin/alginate	1.5/0.75	1389.67 ± 161.64 ^a^	68.44 ± 6.02 ^b^	0.65 ± 0.05 ^ab^
5	Pectin/alginate	3/0.75	1591.00 ± 354.77 ^a^	80.07 ± 2.60 ^a^	0.12 ± 0.02 ^d^

Values are expressed as the mean ± standard deviation (n = 3). Values in the same column followed by different lowercase letters were significantly different according to Tukey’s test at *p* ≤ 0.05.

**Table 3:** Retention profile and encapsulation efficiency of gallic acid microencapsulation.

Wall Material	Retention Efficiency (%)	Encapsulation Efficiency (%)
Alginate 3%	45.84 ± 1.66 ^e^	79.2 ± 3.90 ^c^
Pectin 3%	66.52 ± 3.22 ^b^	84.87 ± 3.13 ^bc^
Pectin/alginate 1.5/1.5%	59.20 ± 1.94 ^c^	89.09 ± 1.21 ^ab^
Pectin/alginate 1.5/0.75%	52.34 ± 3.14 ^d^	82.71 ± 2.70 ^c^
Pectin/alginate 3/0.75%	82.34 ± 2.62 ^a^	90.35 ± 2.21 ^a^

Values are expressed as the mean ± standard deviation (n = 6). Values in the same column followed by different lowercase letters were significantly different according to Tukey’s test at *p* ≤ 0.05.

## Data Availability

The data presented in this study are available on request from the corresponding author.
